# Roles for Golgi Glycans in Oogenesis and Spermatogenesis

**DOI:** 10.3389/fcell.2019.00098

**Published:** 2019-06-07

**Authors:** Ayodele Akintayo, Pamela Stanley

**Affiliations:** Department of Cell Biology, Albert Einstein College of Medicine, New York, NY, United States

**Keywords:** glycosylation, glycans, Golgi, spermatogenesis, oogenesis, fertility

## Abstract

Glycosylation of proteins by N- and O-glycans or glycosaminoglycans (GAGs) mostly begins in the endoplasmic reticulum and is further orchestrated in the Golgi compartment via the action of >100 glycosyltransferases that reside in this complex organelle. The synthesis of glycolipids occurs in the Golgi, also by resident glycosyltransferases. A defect in the glycosylation machinery may impair the functions of glycoproteins and other glycosylated molecules, and lead to a congenital disorder of glycosylation (CDG). Spermatogenesis in the male and oogenesis in the female are tightly regulated differentiation events leading to the production of functional gametes. Insights into roles for glycans in gamete production have been obtained from mutant mice following deletion or inactivation of genes that encode a glycosylation activity. In this review, we will summarize the effects of altering the synthesis of N-glycans, O-glycans, proteoglycans, glycophosphatidylinositol (GPI) anchored proteins, and glycolipids during gametogenesis in the mouse. Glycosylation genes whose deletion causes embryonic lethality have been investigated following conditional deletion using various Cre recombinase transgenes with a cell-type specific promoter. The potential effects of mutations in corresponding glycosylation genes of humans will be discussed in relation to consequences to fertility and potential for use in contraception.

## Introduction

**FIGURE 1:**
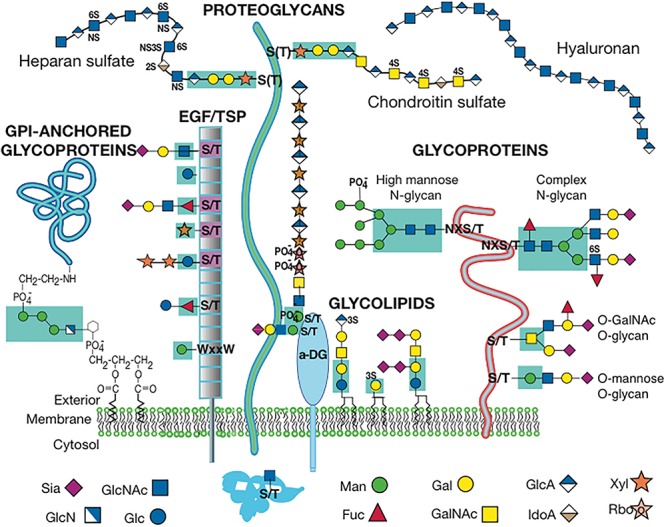
Cell Surface Glycans in Mammals. The diagram depicts one or more glycans from each class of mammalian glycan. The diagram is modified from Figure in Stanley (2016) with permission. Sugar symbols are according to the Symbol Nomenclature for Glycans (Varki et al., 2015).

The mammalian glycome is defined by the genes that encode activities required for the synthesis of glycosylated proteins and lipids. These include genes that encode glycosyltransferases, nucleotide sugar synthases, nucleotide sugar modifiers, nucleotide sugar transporters and glycosidases required to prune glycans during synthesis. Also required for optimal glycosylation are proteins that maintain the structure and environment of the secretory pathway. A summary of the classes of glycan synthesized by mammals is given in Figure 1. The actual complement of glycan structures expressed in the endoplasmic reticulum (ER), in Golgi compartments, at the cell surface, or secreted by a mammalian cell will depend on the glycosylation-related genes that are active in that cell, and that spectrum will likely vary at different stages of development or differentiation. With few exceptions, all glycoproteins, proteoglycans and glycolipids are synthesized in the secretory pathway, although N-glycans and glycosylphosphatidylinositol (GPI) anchors begin their synthesis on the cytoplasmic side of the ER membrane, before an immature glycan is flipped to the luminal side. Maturation continues in the ER and Golgi compartments. Thus, effects on the structure or biochemical environment of the ER and Golgi compartments may affect glycosylation and alter the nature of the glycans produced. The glycans on mature glycoproteins and glycolipids expressed at the cell surface or secreted from a cell are the functional glycans involved in recognition by glycan binding proteins on other cells, and in the extracellular matrices of a tissue. Glycoprotein exceptions to synthesis in the secretory pathway are proteins modified on Ser or Thr by O-GlcNAc in the cytoplasm or nucleus (Zachara et al., 2015), glycogen that is also synthesized in the cytoplasm, and hyaluronan synthesized at the plasma membrane and extruded to the extracellular matrix (Vasconcelos et al., 2013).

A commonly used strategy to determine if a particular glycan or sugar residue is necessary for the development or differentiation of a specific cell type is to inactivate or delete a glycosylation gene. However, this has the drawback that many glycosylated molecules of different functions will be altered. To address functions for glycans in individual glycoproteins, one or more known glycosylation sites can be mutated to preclude their modification. However, relatively few glycans are added at a known amino acid, making this approach arduous, although useful once glycosylation sites have been established. Usually it is important not to eliminate glycosylation of a glycoprotein altogether since this often leads to defective folding, aggregation and degradation and/or inability to exit the ER.

**FIGURE 2:**
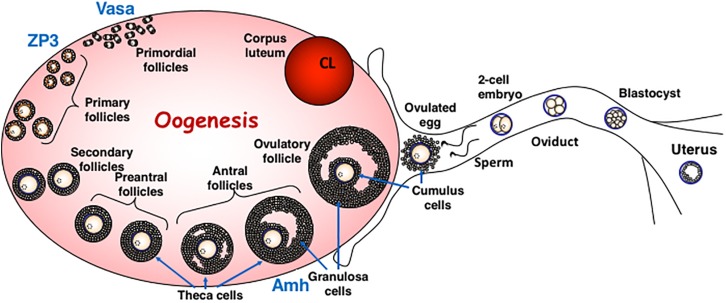
Oogenesis in Mammals. The diagram describes the different stages of oogenesis and identifies the follicular stage in which the ZP3 and Vasa promoters are active in oocytes. Both these promoters are used for oocyte-specific deletion by Cre recombinase. The diagram is modified from (Zhao and Dean, 2002) with permission.

**FIGURE 3:**
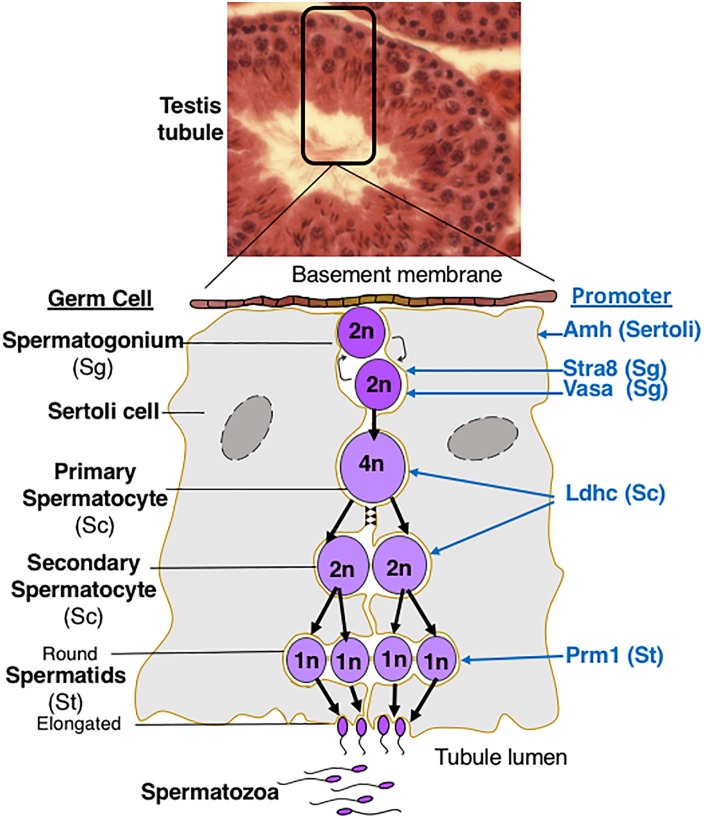
Spermatogenesis in Mammals. The diagram describes germ cell differentiation during spermatogenesis and identifies the germ cell type in which different promoters are active. 2n diploid; 4n, tetraploid; 1n haploid.

**FIGURE 4:**
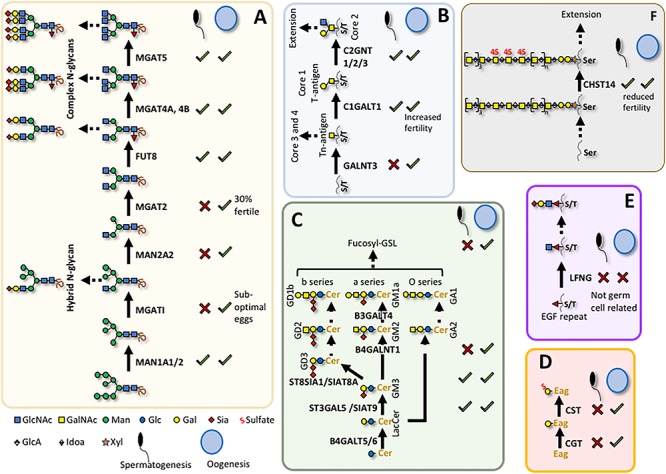
Glycans required for spermatogenesis or oogenesis. Summary of known roles for individual glycans in mouse gametogenesis (see main text). **(A)** N-glycan maturation; **(B)** O-GalNAc core 1 and core 2 glycan synthesis; **(C)** Glycolipid synthesis; **(D)** Seminolipid synthesis; **(E)** O-Fucose glycan synthesis; **(F)** Chondroitin sulfate synthesis. X indicates a defect in male or female gametogenesis and √ indicates no known defect when related gene is deleted; Cer, ceramide; Eag, alkylacylglycerol. Sugar symbols are according to the Symbol Nomenclature for Glycans (Varki et al., 2015).

This review will describe the consequences for oogenesis (Figure 2) and spermatogenesis (Figure 3) of altering glycan synthesis by targeted inactivation of glycosylation genes responsible for the synthesis of Golgi glycans in the mouse. These glycans are identified in Figures 1, 4. Until recently, glycans in the ovary and testis have been investigated by immunohistochemistry and other histochemical assays and by using glycan binding proteins such as plant lectins (Lee and Damjanov, 1984; Wu et al., 1984; Lohr et al., 2010). However, the application of matrix-assisted laser desorption mass spectrometry imaging (MALDI-MSI) has begun to reveal the molecular nature of glycans along with their location in complex tissues (Drake et al., 2018).

## Golgi Glycans Important for Oogenesis in Mammals

Deletion or inactivation of glycosyltransferases that function in the Golgi compartment may have profound or relatively mild effects (Stanley, 2016). For example, inactivation of the *Mgat1* gene which blocks synthesis of all complex and hybrid N-glycans, leads to embryonic lethality (Ioffe and Stanley, 1994; Metzler et al., 1994). By contrast, inactivation of *Mgat5*, which prevents the addition of a single β1,6 branch of complex N-glycans, has much milder consequences (Dennis et al., 2002; Partridge et al., 2004). Conditional deletion of *Mgat1* in oocytes using the ZP3-Cre transgene (Lewandoski et al., 1997) was used to identify roles for complex and hybrid N-glycans in differentiation of oocytes from the primordial follicle stage (Figure 2). Loss of MGAT1 and hybrid and complex N-glycans allowed progression of mutant oocytes to ovulation and fertilization (Shi et al., 2004). Heterozygous embryos from mutant egg and wild type sperm give rise to embryos that develop normally. *Mgat1* null oocytes fertilized by *Mgat1* null sperm also give *Mgat1*[-/-] embryos that develop to ∼E9.5 as observed in a global knockout (Ioffe and Stanley, 1994; Metzler et al., 1994). However, closer examination revealed that females with *Mgat1* null oocytes have ∼25% more empty deciduae than control females, and the zona pellucida of mutant oocytes is very thin. Zona pellucida (ZP) glycoproteins lacked complex N-glycans showing that deletion of *Mgat1* was efficient. While all embryos from *Mgat1* null oocytes implant, not all continue to develop, suggesting that a proportion of oocytes do not give rise to fully functional embryos. Defective oogenesis became more apparent when females were stimulated to ovulate by hormonal treatment. Females producing *Mgat1* null oocytes gave ∼50% fewer ovulated eggs, even though they generated equivalent numbers of antral follicles to controls. In addition, embryos arising from *Mgat1* null eggs were slow in developing through the early stages of embryogenesis prior to implantation. Thus, MGAT1 and complex N-glycans (Figure 4A) are required for the development of fully competent oocytes and ovulated eggs. In addition, these experiments clearly demonstrated that complex or hybrid N-glycans are not required for sperm binding or fertilization. Further investigations revealed that oocytes lacking MGAT1 have aberrant development of preovulatory follicles (retarded folliculogenesis), and structural alterations of the cumulus in cumulus-oocyte complexes (COC; Williams and Stanley, 2009). The reason for sub-optimal development of oocytes that lack MGAT1 may be related to a reduction in growth factor signaling since complex N-glycans regulate the retention of growth factor receptors at the cell surface, and thereby regulate signaling duration (Boscher et al., 2011).

Whole body deletion of *Man2a1* which encodes an alpha-mannosidase II that acts after MGAT1 to prepare the substrate of MGAT2 (Figure 4A), does not result in obvious defects in female fertility (Chui et al., 1997). Global deletion of both *Man2a1* and *Man2a2* (previously called alpha-mannosidase IIX) causes embryonic or perinatal lethality (Akama et al., 2006) and oogenesis in conditional mutants has not been examined. *Mgat2* null mice also die perinatally (Ye and Marth, 2004) in an inbred mouse model, and only 30% of the surviving females in an outbred mouse background were fertile. However, no further description of the basis for the fertility defect, and no targeted deletion in oocytes have been reported. The sugar added after GlcNAc to complex N-glycans in the *trans* Golgi compartment is Gal (Figure 4A). Deletion of *B4galt1* also causes perinatal lethality in one mouse strain (Lu et al., 1997) but not in another (Asano et al., 1997). Females lacking B4GALT1 were fertile but had a slight reduction in litter size when compared to wild type (Asano et al., 1997).

The deletion of another Golgi glycosyltransferase by ZP3-Cre that affects oogenesis gives rise to a phenotype distinct from the loss of *Mgat1*. The glycosyltransferase C1GALT1 (also known as T synthase) transfers Gal to GalNAc attached at Ser/Thr residues, and initiates the synthesis of core 1 and 2 O-GalNAc glycans in the Golgi (Figures 1, 4B). Deletion of *C1galt1* is embryonic lethal at ∼E13.5 (Xia et al., 2004). Conditional deletion by ZP3-Cre in primary follicles (Figure 2) is efficient and strikingly gives rise to an increase in the number of eggs and pups produced by females that lack C1GALT1 in oocytes (Williams and Stanley, 2008). The increase of ∼30–50% in ovulated eggs leads to sustained increases in litter size. While follicular development is enhanced, there is no increase in apoptosis of mutant oocytes (Williams and Stanley, 2008). In addition, oocytes lacking C1GALT1 generate more multiple oocyte follicles (MOF) at late stages of folliculogenesis. The formation of MOFs may be due to the compromised follicular basal lamina apparent in follicles containing *C1galt1* null oocytes (Christensen et al., 2015; Grasa et al., 2016). In fact, robust fusion of mutant follicles is observed in *in vitro* cultures containing follicles with *C1galt1* mutant oocytes, whereas no MOF arose in cultured control oocytes (Christensen et al., 2017). The enhanced folliculogenesis and fertility in females with oocytes lacking C1GALT1 reflects the enhanced sensitivity of follicles containing mutant oocytes to stimulation by follicle stimulating hormone (FSH), reduced apoptosis and altered BMP15-GDF9 signaling (Grasa et al., 2015).

Interestingly, when both *Mgat1* and *C1galt1* were deleted by ZP3-Cre, the results were significantly worse for folliculogenesis, ovulation and fertility than deletion of either one alone (Williams and Stanley, 2011). Removing N-glycans and core 1 and 2 O-GalNAc glycans together abolishes the high fertility phenotype observed in *C1galt1* mutant females and worsens the folliculogenesis defects observed in *Mgat1* null mice, resulting in an overall reduction in egg production and fertility. Nevertheless, eggs lacking both complex and hybrid N-glycans as well as core 1 and 2 O-glycans (and therefore all Gal residues on N- and O-GalNAc glycans) were fertilized, showing that all these glycans are dispensable for fertilization. However, follicle production was greatly reduced, as were the number of ovulated double mutant eggs, and females had, at most, one litter (Williams et al., 2007; Grasa et al., 2012). The extracellular matrix of the cumulus in the cumulus-oocyte complexes (COCs) with double mutant oocytes was also altered (Lo et al., 2018). Ultimately, the combined inactivation of *Mgat1* and *C1galt1* in oocytes led to premature ovarian failure (POF), a novel model for investigation of this syndrome in human premature ovarian insufficiency (POI; Williams and Stanley, 2011; Grasa et al., 2016). *In vitro* culture of double mutant follicles has shown that a subset retains the potential to develop to antral follicles *in vitro* if obtained prior to the development of POF (Kaune et al., 2017). In summary, the generation of complex or hybrid N-glycans as well as core 1 and 2 O-GalNAc glycans in the Golgi is essential for fully functional oogenesis and the production of a complete complement of developmentally-competent eggs and embryos.

Another class of O-glycans is found only on Ser/Thr of epidermal growth factor-like (EGF) repeats at specific consensus sequences (Figure 1; Haltiwanger et al., 2017). Notch receptors and canonical Notch ligands are highly modified by these O-glycans because of the large number of EGF repeats in their extracellular domains. O-fucose, O-glucose and O-GlcNAc residues are transferred to proteins with EGF repeats in the ER by POFUT1, POGLUT1 and EOGT respectively, and further elongated in Golgi compartments (Takeuchi and Haltiwanger, 2014). Deletion of the initiating glycosyltransferase genes is embryonic lethal for O-fucose and O-glucose glycans but not for O-GlcNAc glycans (Varshney and Stanley, 2018). Female mice lacking secretory pathway O-GlcNAc glycans are fertile. Surprisingly, females with ZP3-Cre-mediated deletion of *Pofut1* leading to the loss of all O-fucose glycans in oocytes, also have normal fertility, and thus apparently normal oogenesis (Shi et al., 2005), despite proposed roles for Notch signaling in oogenesis (Xu and Gridley, 2013; Feng et al., 2014). By contrast, the global deletion of *Lfng*, a Golgi GlcNAc-transferase which adds GlcNAc to O-fucose-modified EGF repeats, leads to infertility in female mice due to defective follicular development and meiotic maturation of oocytes (Hahn et al., 2005). Because the loss of O-fucose on EGF repeats has no effect on female fertility, the *Lfng* null phenotype must not be due to defect(s) in oocytes but in some other cell(s) required for normal oogenesis (Figure 4E). No effects on female fertility were reported in mice lacking the related GlcNAc-transferases *Mfng* or *Rfng* (Moran et al., 2009).

Only in a few cases have specific roles in oogenesis and/or female fertility been reported for mice with disrupted synthesis of glycosaminoglycans (GAG), glycosylphosphatidylinositol (GPI) anchors or glycolipids. While female mice lacking dermatan-4-O-sulfotransferase 1 (CHST14) are fertile when crossed with wild type males and *Chst14* null males are also fertile when crossed to wild type females, homozygous null females crossed with homozygous null males give no progeny (Akyuz et al., 2013; Figure 4F). GAGs as well as glycolipids and other glycans of glycoproteins are main components of the extracellular matrix and, it is known that the composition and structure of the extracellular matrix is critical for the development of follicles and eggs in the ovary (Nagyova, 2018). The extracellular matrix and COC are significantly compromised in mice with oocytes lacking MGAT1 (Williams and Stanley, 2009) or both MGAT1 and C1GALT1 (Lo et al., 2018). ZP3 promoter-directed conditional deletion of *Piga*, the enzyme that initiates GPI-anchor synthesis in the endoplasmic reticulum (ER), gives rise to females in which folliculogenesis and egg production appear normal but mutant eggs lacking PIGA and thus GPI-anchored proteins at the cell surface are unable to fuse with sperm (Alfieri et al., 2003). One reason for this may be the absence of the GPI-anchored protein JUNO at the egg membrane. JUNO is essential for sperm binding to the egg via its ligand Izumo1 (Bianchi et al., 2014).

Despite the indications from mouse mutants that fertility would be expected to be affected in women with altered glycosylation, a literature search did not reveal known genetic bases associated with glycosylation genes that correlate with defective oogenesis, fertility or POI in humans. Nor do the results in mice to date suggest that disruption of glycosylation would be an effective female contraceptive.

## Golgi Glycans Important for Spermatogenesis in Mammals

Spermatogenesis in mammals is a multi-step differentiation process in which spermatogonia (Sg), spermatocytes (Sc), spermatids (St) and ultimately spermatozoa are generated in close contact with Sertoli cells (Figure 3). The first Golgi glycosylation gene knockout in the mouse to reveal a role for N-glycans in spermatogenesis was global deletion of *Man2a2* (Akama et al., 2002; Fukuda and Akama, 2003), an alpha-mannosidase II which removes two mannose residues from the N-glycan product of MGAT1 and generates the substrate for MGAT2 (Figure 4A). The protein is enriched in germ cells except spermatogonia and condensing spermatids, and is not prominent in somatic cells. The N-glycan product of alpha-mannosidase IIx, detected in testis sections using the lectin *Griffonia simplicifolia* (GSA), was detected on spermatogenic cells (except spermatogonia) but not somatic cells (Sertoli, Leydig), suggesting that MAN2A2 activity is mainly restricted to germ cells in the seminiferous tubule (Akama et al., 2002). Germ cells of the *Man2a2* whole body knockout form multi-nuclear cells (MNC or syncytia) and the mice are infertile. Complex N-glycans are absent based on the lack of GSA binding to germ cells. Hybrid N-glycans are expected to be present after deletion of *Man2a2* (Figure 4A). Thus, neither hybrid nor oligomannose N-glycans on glycoproteins were able to support spermatogenesis or the production of normal numbers of mature sperm. Testes from the *Man2a2* global knockout were smaller, contained fewer spermatids and produced immature sperm. Adhesion of *Man2a2* mutant germ cells to Sertoli cells was reduced and a N-glycan terminating in GlcNAc was proposed to be necessary for germ-Sertoli cell adhesion. Global deletion of the other alpha mannosidase II encoded by the *Man2a1* gene does not affect viability or male fertility, but whole body knockout of both *Man2a1* and *Man2a2* is neonatal lethal (Akama et al., 2006; Hato et al., 2006). Unusual hybrid N-glycans observed in tissues from E15.5 *Man2a1/Man2a2* double knockout embryos (Hato et al., 2006) were not reported in germ cells lacking MAN2A2 (Akama et al., 2002). Disruption of the *Man1a2* gene that encodes an alpha mannosidase involved in the pruning of oligomannose N-glycans, is perinatal lethal (Tremblay et al., 2007), and conditional deletion in germ cells has not been reported.

A marked spermatogenic phenotype is obtained when *Mgat1* is specifically deleted in spermatogonia using Stra8-iCre (Batista et al., 2012). In males with germ cells lacking both complex and hybrid N-glycans, testes are small, MNCs are very prominent in testis tubules, apoptosis is increased, and there are no mature sperm in epididymis. Investigation into potential mechanisms of the block in spermatogenesis identified ERK1/2 signaling as markedly reduced in the absence of MGAT1 (Biswas et al., 2018). A physiological inhibitor of MGAT1 termed GnT1IP or MGAT4D, and expressed most highly in male germ cells (Huang and Stanley, 2010; Huang et al., 2015), is being investigated as a potential regulator of complex N-glycan production during spermatogenesis. Whole body disruption of *Mgat2* gene expression is lethal in the early days postpartum in inbred mice. On an “outbred” background, the few survivors have spermatogonia and spermatocytes but no round spermatids or mature spermatozoa in the lumen of the seminiferous tubules (Wang et al., 2002). Inactivation of *Fut8* encoding the fucosyltransferase responsible for N-glycan core fucosylation, induces early postnatal death (Wang et al., 2005) and no targeted deletion in germ cells has been reported.

The absence of core 1 and 2 O-glycans due to deletion of *C1galt1* by Stra8-iCre, has no effect on spermatogenesis (Batista et al., 2012). Interestingly, neither does deletion of *Pofut1*, and thus the absence of O-fucose glycans does not disrupt spermatogenesis (Batista et al., 2012). Deletion of *Pofut1* in Sertoli cells using Amh-Cre also has no deleterious effect on spermatogenesis (Hasegawa et al., 2012). Nor does the absence of NOTCH1 in germ cells (Batista et al., 2012). However, global deletion of the Golgi GlcNAc-transferase encoded by *Lfng*, which acts after POFUT1, gives rise to a defective rete testis (Hahn et al., 2009). This results in few sperm in the epididymis, although spermatogenesis progresses normally. Since this was not observed in *Pofut1* conditional germ cell knockout males, the *Lfng* knockout rete testis phenotype is probably due to defective development of non-germ cells of the testis. Global deletion of *B4galt1* leads to delayed spermatogenesis in the few males that do not die perinatally (Lu et al., 1997). Seminiferous tubules are smaller and spermatids are fewer in *B4galt1* null testes. However, fertility and viability are not affected in *B4galt1* null males on a different genetic background (Nishie et al., 2007).

In summary, hybrid and complex N-glycans (Figure 4A) are required in germ cells for spermatogenesis to progress to mature sperm but core 1 and core 2 O-GalNAc glycans (Figure 4B) or O-fucose glycans (Figure 4E) are dispensable. Interestingly, however, some GalNAc residues on Ser/Thr, potentially unmodified further or extended to make core 3 or core 4 O-GalNAc glycans (Brockhausen and Stanley, 2017), are required for spermatogenesis. Deletion of a GalNAc transferase termed *Galnt3*, which is one of approximately 20 polypeptide GalNAc transferases that initiate the synthesis of O-GalNAc glycans in the Golgi, leads to disrupted spermatogenesis and male infertility (Ichikawa et al., 2009; Miyazaki et al., 2013). GALNT3 localizes to early and medial Golgi compartments of spermatids and spermatocytes (Miyazaki et al., 2013). GALNT3 seems to be important for O-GalNAc addition to proteins in testis because its deletion can be easily detected by lectins that bind to GalNAc-O-Ser/Thr. Deletion leads to defects in acrosome formation, increased apoptosis in the seminiferous tubule, reduced production of sperm, and oligoasthenoteratozoospermia (rare and immotile sperm, deformed round head spermatozoa). A related member of the GALNT family termed GALNTL5, which is lacking a portion of the usual GALNT C-terminal domain and has no known GalNAc-transferase activity, is exclusively expressed in testis germ cells. The protein is detected in the cytoplasm of round spermatids, around the acrosome of elongated spermatids, and in the neck region of spermatozoa. Haploinsufficiency causes spermatogenic defects in the mouse due to immotile spermatozoa (Takasaki et al., 2014). Heterozygosity of *GALNTL5* also leads to immotile sperm in men (Hagiuda et al., 2019). It will be interesting to know the function of this novel glycosyltransferase-like protein.

Glycolipids synthesized in the Golgi (Figure 4C) are also important in spermatogenesis. This was initially noted in mice lacking GM2/GD2 synthase (B4GALNT1) which synthesize only Glc-ceramide (Glc-Cer), Lactosyl-Cer, GM3 and GD3 Golgi glycolipids (Takamiya et al., 1998). Male mice lacking B4GALNT1 are sterile and do not produce sperm. Spermatogenesis proceeds to the stage of round spermatids but maturing spermatids fuse into MNCs and sperm are not produced. Investigations of Glycosphingolipids (GSL) in wild type testes compared to *Galnt1*[-/-] testes identified novel fucosylated neutral and monosialylated GSL with very long polyenoic ceramides termed FGSL (Sandhoff et al., 2005). However, the absence of sialylated GSL following deletion of GM3 synthase (ST3GAL5/SIAT9) or GD3 synthase (ST8SIA1) and therefore the loss of sialylated FGSL has no effect on male fertility (Kawai et al., 2001; Yamashita et al., 2003, 2005). Thus, the neutral Golgi FGSL found in germ cells but not Leydig or Sertoli cells (Sandhoff et al., 2005) appear to be essential for spermatogenesis and male fertility. This result suggests that O-series gangliosides are sufficient for male spermatogenesis and sialic acid residues are not necessary on these glycolipids during germ cells maturation (Figure 4C). The loss of seminolipid (HSO3-3-monogalactosylalkylacylglycerol or SO4-Gal-Eag; Figure 4D) by deletion of the galactosyltransferase CGT also disrupts spermatogenesis (Fujimoto et al., 2000). *Cgt* is expressed in later-stage spermatocytes and deletion does not disrupt the formation of spermatogonia or early spermatocytes. However, mutant males have no spermatids or sperm. Deletion of *Cgt* precludes the addition of Gal to the precursor of seminolipid (Eag). However, subsequent deletion of the sulfotransferase *Cst* gives a similar phenotype to deletion of *Cgt* (Honke et al., 2002) showing that Gal-Eag is not able to fulfill the functions of SO4-Gal-Eag in testis. *Cst* is expressed in germ cells and germ cells of *Cst* null males do not proceed through the first meiotic division (Honke et al., 2002; Zhang et al., 2005).

The GPI-anchor on the subset of GPI-anchored proteins in testis is initiated on the ER membrane and additional sugars are added in the Golgi compartment (Ferguson et al., 2017; Figure 1). While numerous GPI-anchored proteins are important in the formation of sperm, effects specific to GPI anchor Golgi glycosyltransferases have not been reported. The ER resident protein PGAP1 (post GPI attachment to proteins 1) is a GPI inositol-deacylase involved in the maturation of the GPI-anchor by removing palmitate from inositol. A whole body deletion of the gene does not affect spermatogenesis but induces a severe reduction in male fertility. The ability of sperm to ascend into the oviduct and to attach to the zona pellucida of the oocyte is severely impaired (Ueda et al., 2007). Inactivation of the *Piga* gene which blocks the formation of GPI anchors altogether is embryonic lethal, but chimeric mice generated by injecting *Piga* null ES cells into wild type blastocysts allowed mice with *Piga* null and wild type cells to be born and spermatogenesis to be investigated (Lin et al., 2000). Chimeric males were sterile. While spermatogenesis was markedly reduced, sperm were made and sperm lacking PIGA were detected in the epididymis. However, *Piga* null sperm were not transmitted.

In searches for connections between reduced fertility in men and defective glycosylation, only one mutation in a putative glycosyltransferase gene has been identified - GALNTL5 (Hagiuda et al., 2019). However, many more glycosyltransferase defects may ultimately be tied to altered spermatogenesis and fertility in men if results from gene deletions in mouse are a guide. In addition, the mechanisms of defective spermatogenesis in mice vary depending on the glycosylation pathway affected. Glycosyltransferases whose loss appears to spare spermatogonia and spermatocytes but result in fused spermatids and no mature sperm are good candidates for reversible contraception in men. Thus, MGAT1 (Figure 4A) and B4GALNT1 (Figure 4C) make good inhibitor targets for development as male contraceptives.

## Conclusion and Future Directions

Although relatively few mice with germ-cell specific deletion or inactivation of a glycosylation gene have been investigated, it is clear from these experiments that different classes of glycans are required for different aspects of oogenesis and spermatogenesis in mammals. Thus, complex N-glycans are necessary for optimal oogenesis and the production of fully functional ovulated eggs, and they are essential for the production of sperm. This is not due to general defects in protein folding or degradation since MGAT1 acts in the medial Golgi, long after glycoproteins have exited the ER. By contrast, core 1 and core 2 O-GalNAc glycans are not required for spermatogenesis but are important for the regulation of oogenesis and female fertility and for protection from POF. O-fucose glycans are dispensable for both oogenesis and spermatogenesis when deleted in germ cells. The requirement for LFNG in both female and male fertility is predicted to be due to its functions in non-germ cells. Complex glycolipids are necessary for spermatogenesis but appear to be dispensable for oogenesis. Glycosaminoglycans have not been sufficiently investigated to know if they may be important in fertility. Inhibiting synthesis of all GPI anchors does not prevent sperm from being produced but mutant sperm are not transmitted. In females, the absence of GPI anchors impairs the ability of oocytes to fuse with sperm. There is only one human mutation in a putative O-GalNAc-transferase, GALNTL5, which is associated with infertility. However, other genetic bases of defective oogenesis, spermatogenesis or fertility are sure to be detected in humans based on results to date in mutant mice. The sensitivity of spermatogenesis to defects in all these pathways clearly indicates the potential to give rise to idiopathic cases of infertility.

## Author Contributions

PS and AA wrote the manuscript. PS modified the figures. AA designed Figures 3, 4.

## Conflict of Interest Statement

The authors declare that the research was conducted in the absence of any commercial or financial relationships that could be construed as a potential conflict of interest.
